# Trophoblast derived extracellular vesicles specifically alter the transcriptome of endometrial cells and may constitute a critical component of embryo-maternal communication

**DOI:** 10.1186/s12958-021-00801-5

**Published:** 2021-07-21

**Authors:** Kasun Godakumara, James Ord, Freddy Lättekivi, Keerthie Dissanayake, Janeli Viil, Nageswara Rao Boggavarapu, Omid R Faridani, Kersti Jääger, Agne Velthut-Meikas, Ülle Jaakma, Andres Salumets, Alireza Fazeli

**Affiliations:** 1 Department of Pathophysiology, Institute of Biomedicine and Translational Medicine, Faculty of Medicine, Tartu University, Tartu, Estonia; 2 Institute of Veterinary Medicine and Animal Sciences, Estonian University of Life Sciences, Tartu, Estonia; 3 Division of Obstetrics and Gynaecology, Department of Women’s and Children’s Health, Karolinska Institute, Karolinska University Hospital, S-171 76 Stockholm, Sweden; 4 Garvan Institute of Medical Research, Sydney, Australia; 5 Lowy Cancer Research Centre, School of Medical Sciences, University of New South Wales, Sydney, Australia; 6 Competence Centre On Health Technologies, Tartu, Estonia; 7 Department of Chemistry and Biotechnology, School of Science, Tallinn University of Technology, Tallinn, Estonia; 8 Department of Obstetrics and Gynaecology, Institute of Clinical Medicine, University of Tartu, Tartu, Estonia; 9 Institute of Genomics, University of Tartu, Tartu, Estonia; 10 Academic Unit of Reproductive and Developmental Medicine, Department of Oncology and Metabolism, Medical School, University of Sheffield, Sheffield, UK

**Keywords:** Embryo-maternal communication, Extracellular vesicles, miRNA signalling, RNA-sequencing

## Abstract

**Background:**

The period of time when the embryo and the endometrium undergo significant morphological alterations to facilitate a successful implantation—known as “window of implantation”—is a critical moment in human reproduction. Embryo and the endometrium communicate extensively during this period, and lipid bilayer bound nanoscale extracellular vesicles (EVs) are purported to be integral to this communication.

**Methods:**

To investigate the nature of the EV-mediated embryo-maternal communication, we have supplemented trophoblast analogue spheroid (JAr) derived EVs to an endometrial analogue (RL 95–2) cell layer and characterized the transcriptomic alterations using RNA sequencing. EVs derived from non-trophoblast cells (HEK293) were used as a negative control. The cargo of the EVs were also investigated through mRNA and miRNA sequencing.

**Results:**

Trophoblast spheroid derived EVs induced drastic transcriptomic alterations in the endometrial cells while the non-trophoblast cell derived EVs failed to induce such changes demonstrating functional specificity in terms of EV origin. Through gene set enrichment analysis (GSEA), we found that the response in endometrial cells was focused on extracellular matrix remodelling and G protein-coupled receptors’ signalling, both of which are of known functional relevance to endometrial receptivity. Approximately 9% of genes downregulated in endometrial cells were high-confidence predicted targets of miRNAs detected exclusively in trophoblast analogue-derived EVs, suggesting that only a small proportion of reduced expression in endometrial cells can be attributed directly to gene silencing by miRNAs carried as cargo in the EVs.

**Conclusion:**

Our study reveals that trophoblast derived EVs have the ability to modify the endometrial gene expression, potentially with functional importance for embryo-maternal communication during implantation, although the exact underlying signalling mechanisms remain to be elucidated.

**Supplementary Information:**

The online version contains supplementary material available at 10.1186/s12958-021-00801-5.

## Background

Embryo implantation is a crucial step in human reproduction where the embryo initiates physical contact with maternal tissue. The embryo first loosely attaches itself to the endometrial epithelial lining and then proceeds to invade the uterine stromal cell layer [1]. The endometrial epithelium and the underlying stromal cells go through remarkable alterations to facilitate the process of implantation. Most of the physiological and biochemical changes happening in the vicinity of embryo attachment site are localized to the implantation site only, giving rise to the theory that the embryo itself directs the changes required for implantation in the endometrium [2, 3]. Subsequently, the mechanisms of endometrial regulation arising from the embryo have emerged as a subject of intense scientific investigation.

One of the hypotheses being investigated in this regard is the theory of embryo-maternal cross-talk which posits that the embryo and the endometrium undergo a complex set of “negotiations” prior to and during apposition, the first step of implantation, in the time period known as the “window of implantation” (WOI) [4, 5]. The cross-talk prepares both the embryo and the endometrium for a successful implantation by inducing specific biochemical alterations in the epithelial cells and the underlying uterine architecture [6, 7]. Inadequate intercellular dialog would result in implantation failure, which is also the main cause of unsuccessful attempts in assisted reproduction [8–10]. Evidence of embryo-maternal cross-talk is reported by a number of studies focusing on different modes of signalling. For example, hormonal regulation of endometrial tissues is a well-known phenomenon [11–13], There is also evidence of cytokine based regulation of endometrial tissue during the WOI [14–17] and direct protein–protein embryo-maternal interactions [18]. However, as embryo implantation is a temporally and spatially complex process, it likely involves several other mechanisms that are less known.

One of the alternative methods of communication under consideration is extracellular vesicle (EV) mediated communications. EVs are a heterogeneous group of membrane bound small (diameter of 20 – 1000 nm) spherical biological structures known to be secreted by every known cell type. Although they were initially considered ‘cellular garbage’ of little interest, in the recent years the concept of EVs as means of intercellular communication has gained traction.

EV-mediated communication may involve diverse mechanisms. They carry an extensive surface proteome and possess a large surface to volume ratio. Mediated by these surface bound molecules, EVs take part in a multitude of molecular interactions with the target cell membrane. These exchanges form a connection between the EV source and the target cell accomplishing a plethora of physiological functions [19–21]. Another well-known method of EV based communication concerns the EV cargo. EVs are known to carry a diverse cargo of RNAs, lipids and proteins which are protected from the destructive enzymesin the extracellular space [22–24]. EVs are taken up by target cells via various mechanisms such as macropinocytosis, phagocytosis, and lipid raft–mediated internalization [25]. The cargo molecules are then released into the target cell’s cytoplasm. Various instances of intercellular communication, including embryo-maternal communication, are reported to involve EVs [26–29].

In an earlier study employing an in vitro model of embryo-maternalcommunication, we showed that labelled RNA can be transferred remotely from trophoblast cells to endometrial cells via EVs [26]. In the current study, using the same in vitro system, we investigated the hypothesis that embryo-derived EVs are capable of specifically altering the transcriptomic profiles of endometrial cells. We analysed the RNA cargo of trophoblast EVs and EVs from a cell line of a non-reproductive origin (HEK-293, kidney origin). Co-incubation with trophoblast derived EVs induced substantial changes to the transcriptomic profile of endometrial cells, while no such alterations were observed after HEK EV co-incubation with endometrial cells. These data support the hypothesis that trophoblast EVs specifically interact with endometrial cells and may affect the gene expression in endometrial cells partially through their molecular cargo.

Because of the well-known ethical dilemmas of using human embryos for experimentation, we have used an in vitro model of the pre-implantation microenvironment in the current study. Human choriocarcinoma cell line JAr in 3D spheroidal form was used as an analogue for the trophoblast cells and the RL95-2 cell line was used as an analogue for the mid-secretary/WOI receptive endometrium.

## Methods

### Cell culture and spheroid formation

The human endometrial adenosquamous carcinoma cell line (RL95-2) was obtained from American Type Culture Collection (ATCC CRL-1671, Teddington, UK). RL95-2 was cultured in Dulbecco’s Modified Eagles Medium (DMEM 12-604F, Lonza, Verviers, Belgium) supplemented with 1% Penicillin/Streptomycin (P/S, Gibco™ 15,140,122, Bleiswijk, Netherlands), 5 μg/ml Insulin (human recombinant insulin, Gibco, Invitrogen, Denmark), 1% L-glutamine (Sigma, 59202C, Saint Louis, USA) and 10% fetal bovine serum (FBS, Gibco™, 10,500,064) at 37 °C in 5% CO2 conditions.

The human choriocarcinoma cell line (JAr) from the first trimester trophoblasts was acquired from ATCC® (HTB-144 ™, Teddington, UK). JAr cells were cultured in a T75 flask in RPMI 1640 media (Gibco, Scotland) supplemented with 10% FBS, 1% L-glutamine and 1% P/S at 5% CO_2_ in 37 °C. At confluency, JAr cells were washed with Dulbecco’s phosphate-buffered saline without Ca^+2^ and Mg^+2^ (DPBS, Verviers, Belgium), harvested using trypsin–EDTA (Gibco® Trypsin, New York, USA) and pelleted by centrifugation at 250 g for 5 min. 1 × 10^6^ cells/ml were cultured in 5 ml of supplemented RPMI 1640 medium in 60 mm Petri dishes at 5% CO_2_ in 37 °C. The cells were kept on a gyratory shaker (Biosan PSU-2 T, Riga, Latvia), set at 295 rotations per min (rpm) for 18 h. The multicellular spheroids were used to mimic trophoblast cells in vitro.

The human embryo kidney (HEK) 293 T cell line (acquired from ATCC®, CRL-3216™, Teddington, UK) was cultured in DMEM supplemented with 10% of heat-inactivated FBS (Gibco) and 1% L-glutamine (Sigma). All cells were grown in T75 flasks at 37 °C in 5% CO_2_conditions. The media was changed every second day until confluence of the cells. One million cells were counted with a haemocytometer and cultured overnight on a gyratory shaker to form multicellular spheroids as described above.

### Preparation of EV depleted medium

EV depleted FBS was produced using the ultrafiltration method described by Kornilov et al*.* in 2018 [30]. Briefly, the FBS was filtered using Amicon ultra-15 centrifugal filters (100 kDa, MERCK KGAA, Darmstadt, Germany) at 3,000 g for 55 min. This method removed 90% of the nanoparticles from the FBS. The filtered FBS was used as a 10% supplementation for all the cell type specific complete culture media described above to prepare the EV depleted complete media.

### EVs purification and characterization

EVs were harvested from conditioned media of spheroid culture of both the JAr and HEK293 cell lines. Conditioned media was centrifuged at 400 g for 10 min. The supernatant was again centrifuged at 4,000 g for 10 min and thereafter at 20,000 g for 15 min to get rid of cell debris and apoptotic bodies. To isolate EVs, conditioned media was concentrated to 500 µl with Amicon® Ultra-15 centrifugal filter devices (10 kDa cut-off). RNase inhibitor (1u/µl, Recombinant RNasin®, Promega corp., 2800, Woods Hollow Road, Madison, WI) was added to conditioned media to protect EVs’ RNA during the isolation process. EVs were isolated using size exclusion chromatography (SEC). A cross linked 4% agarose matrix of 90 µm beads were used (Sepharose 4 fast flow™, GE HealthCare Bio-Sciences AB, Uppsala, Sweden) in a 10 cm column. Fractions 7–10 (fraction size 1 ml) were collected. Fractions were concentrated using Amicon® Ultra-15 centrifugal filter devices (10 kDa cut-off). Isolated EVs were characterized following the protocols described elsewhere in details [26, 31]. Briefly, EVs were quantified by nano-particle tracking analysis using ZetaView (Particle Metrix GmbH, Inning am Ammersee, Germany). Surface proteome of the isolated EVs were analysed using western blot for standard EV markers, CD63, CD81 and CD9. Morphology of the EVs was observed using transmission electron microscopy. EV characterization methods were published in our earlier study [26].

### Whole RNA extraction and quality control

Whole RNA was extracted from cells and EVs by TRIzol Reagent and isopropanol precipitation (TRIzol® reagent; Invitrogen). To increase the efficiency of RNA extraction, 20 µg glycogen (UltraPure™ Glycogen, Cat. no. 10814–010, Thermo Fisher Scientific, Bleiswijk, Netherlands) was added to the lysis buffer per sample. The RNA pellet was washed three times by 75% ethanol. Quality and quantity of the extracted RNA samples were analyzed by Bioanalyzer Automated Electrophoresis instrument (Agilent technologies, Santa Clara, CA) using Agilent RNA 6000 Pico kit (Agilent technologies).

### cDNA Library preparation and mRNA sequencing

RNA sequencing libraries were generated using multiplexing capacity of Smart-seq2 methodology with slight modifications [32]. Instead of single cells, 20 ng of total RNA was used for cDNA synthesis and 10 cycles of PCR for pre-amplification. KAPA HiFi DNA polymerase was replaced with Phusion High-Fidelity DNA Polymerase (Thermo Scientific) compatible with the original protocol. 2 μL of diluted cDNA was applied to dual-index library preparation using Illumina Nextera XT DNA Sample Preparation Kit (FC-131–1024). AMPure XP beads (Beckman Coulter) were used for all clean-up steps and for size selection (200–700 bp). All samples were pooled into single library by equal concentration and sequenced on Illumina NextSeq500 using High Output Flow Cell v2.5 (single-end, 75 bp).

### Processing, alignment, and quantification of RNAsequencing(RNAseq) reads

The quality of raw reads was assessed using FASTQC v0.11.8 [33]. Trimmomatic v0.39 was used for read trimming and removal of adaptor sequences, using the following parameters: LEADING:20, SLIDINGWINDOW:4:15, ILLUMINACLIP: *:1:30:15 and MINLEN:25.

Reads were aligned to the hg38 human reference genome. The alignment was performed using HISAT2 [34] with default parameters and with the inclusion of splice site information derived from the corresponding Ensembl *H. sapiens* annotation file (GRCh38.97). The sequencing of EV RNA samples yielded relatively low percentage of reads mapped to the genome. For HEK293 EVs, on average 3.08% of 6,820,518 total alignments were successfully assigned. In case of JAr EVs, 4.48% of 4,672,213 total alignments were successfully assigned on average. For RL95-2 cells treated with JAr EVs, 5,747,968 reads were aligned on average and 32.39% of which were successfully assigned to the genome. Average number of aligned reads and percentage of successfully assigned reads were 5,282,088 (56.84%) in case of untreated RL95-2 cells and 4,974,678 (54.94%) for RL 95–2 cells treated with HEK293 EVs. Gene-level read counts were obtained using featureCounts [35] with default parameters, using the Ensembl *H. sapiens* annotation file (GRCh38.97) for genomic feature annotations. Genes with at least 10 counts for all the samples in at least one of the experimental groups were retained in the analysis for subsequent differential expression testing.

### Differential gene expression analysis

Differential expression (DE) analysis was carried out in R version 3.6.1 using the edgeR package version 3.26.8 [36]. Tagwise dispersion estimates were obtained based on the trended dispersions, and statistical comparisons were performed using a generalized linear model followed by likelihood ratio tests, also accounting for the experiment batch. We considered the differential expression of geneswith a false discovery rate (FDR) ≤ 0.05 to be statistically significant.

Gene set enrichment analysis (GSEA), and pathway over-representation analysis was conducted using the ReactomePA package [37] and Reactome Pathway database annotations [37]. GSEA was used for full gene lists obtained from DE analysis that were ranked by -log10p × log2FC, where p denotes unadjusted p-values and FC the fold-change.

Principal components were calculated using prcomp function from the Stats package and visualized using the ggplot2 package [38]. The pheatmap package [39] was used for heatmap visualization with hierarchical clustering based on Euclidean distance.

### RNA extraction for miRNA sequencing

The EVs (1 × 10^8^ EVs extracted from 10 ml of conditioned medium)were sorted into 100 µl of RLT buffer (Qiagen) and proceeded for RNA extraction. Briefly, 100 µl of RLT buffer with sorted EVs was mixed with 2 µl of pellet paint (Merck Millipore), vortexed briefly, 19 µl of 3 M Sodium Acetate (pH 5.5) and 300 µl of 100% ethanol was added and vortexed briefly and incubated at + 4 °C overnight. The contents were then centrifuged at 16,000 g for 15 min at 4 °C and the supernatant wascarefully discarded without disturbing the pellet. The pellet was washed twice with fresh 1 ml of 80% ethanol and air dried. The pellet was resuspended in 10 µl of RNase free water and stored at -80 °C till further use.

### Small RNA library construction and data analysis

The small RNA transcriptome library was constructed for the EV’s from both the JAr and HEK293 cell lines,as described in the protocol published by Faridani et. al. [40, 41], using 3 µl of extracted whole RNA from EV’s and HEK293cells. The amplified libraries were then purified using AMPure XP beads with 1:1 ratio of sample to beads as per the manufsacturer’s protocol and eluted in 10 µl of RNase free water. DNA quantification was done using Qubit HS DNA analysis (Thermo Scientific) and the DNA library quality control was performed on Bioanalyzer 2100 station (Agilent). 5 ng of DNA from each sample was pooled and sequenced 1 × 100 bp using Illumina NovaSeq platform (National Genomics Infrastructure, SciLifeLab, Sweden).

The initial data analysis was performed on the Partek Flow bioinformatics software (Partek Inc, USA). Briefly, all the fastq files were screened and the contaminating reads from the mitochondrial DNA and ribosomal DNA were removed. Theunique molecular identifiers (UMI’s) were removed from the sequences and appended to the read names for later analysis. Adapters and poly(A) sequences were removed from the reads and the trimmed reads were aligned to human genome Hg38 using Bowtie 2 aligner with a seed length of 10 and seed mismatch of 1nt. Post alignment the UMIs were deduplicated and the reads were quantified to Hg38 miRBase mature miRNAs database, version 22.

### Identification of putative JAr-specific miRNAs and their putative targets in RL95 cells

To identify putative JAr EV-specific miRNAs, we examined miRNA alignment counts from three small RNAseq libraries derived from JAr EVs (total genome-mapped reads: 1,359,431) alongside three derived from HEK293EVs (total genome-mapped reads: 1,912,942). The dataset was filtered to retain miRNAs which were detected in at least 2/3 libraries of either of JAr or HEK293EVs. We subsequently counted the number of miRNAs which were detected above raw counts thresholds of 1, 3, 5, and 10 in the required number of samples. For downstream analysis, miRNAs were considered specific to JAr EVs if they were represented by at least five counts in 2/3 JAr EV libraries while not being detected at all in any of the HEK293EV libraries.

We obtained a list of all predicted target transcripts from miRDB [42]. These were filtered to retain only high-confidence targets (those with a target score of ≥ 90). Using the R package AnnotationDbi [43], REFSEQ transcript IDs were converted to ENSEMBL gene IDs to obtain the list of predicted miR targets at the gene level. We were thus able to identify putative miRNA targets in the RL95-2 gene expression dataset by matching the ENSEMBL IDs. We subsequently counted the number of putative targets within the RL95-2 gene expression dataset that were downregulated, upregulated, and non-DE for each miRNA.

**Fig. 1:**
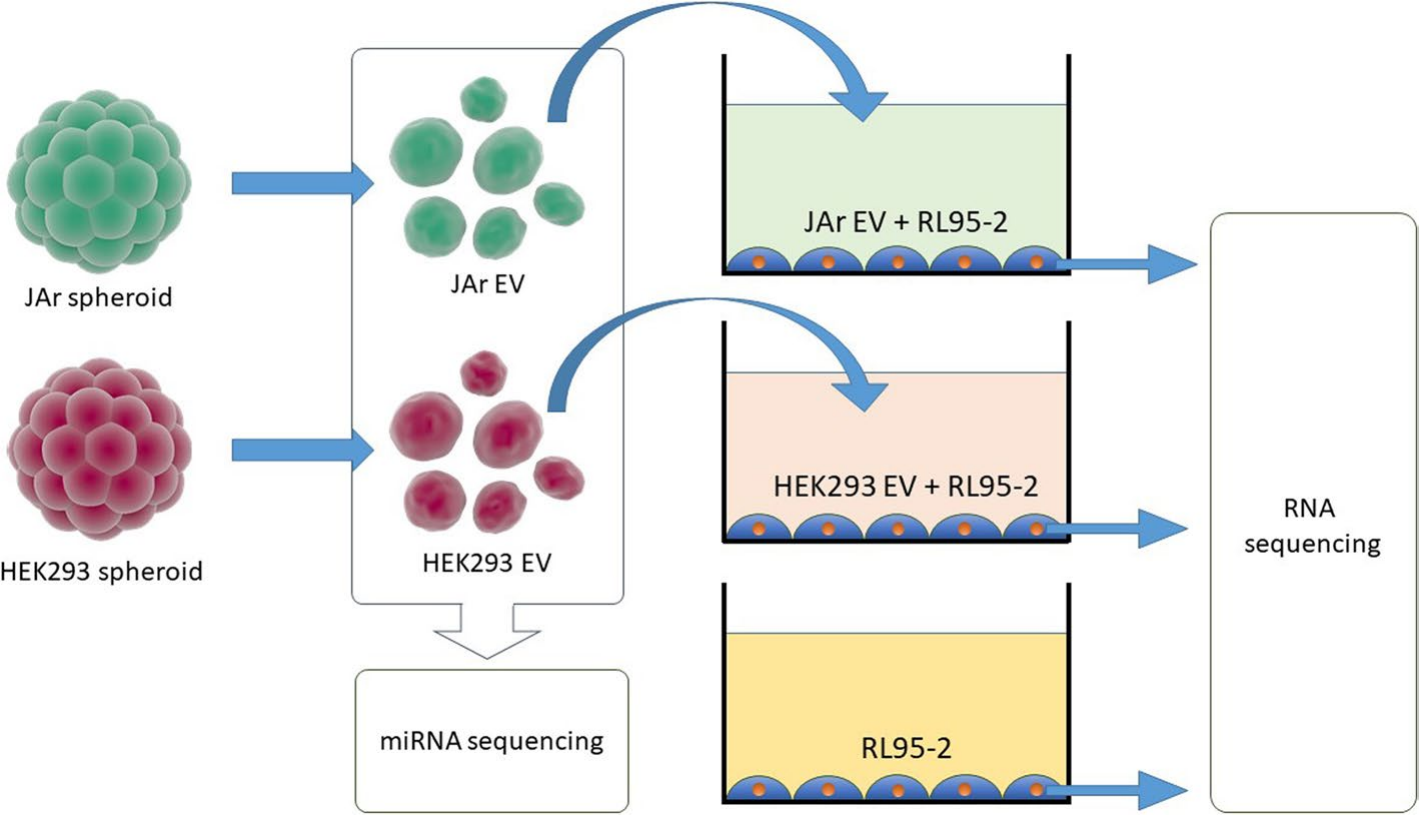
Experimental design of the study. Extracellular vesicles (EV) were isolated from trophoblast (JAr) spheroids and non-trophoblast (HEK293) spheroids. The miRNA cargo of the isolated EVs was explored using miRNA sequencing. EVs were supplemented to the endometrial analogue (RL95-2) cells and the transcriptomic alterations of the RL95-2 cells were investigated using RNA sequencing

Focusing on downregulated putative targets, we then sought to ascertain whether the abundance of a given miRNA from EVs corresponded with the extent of repression of downregulated targets in RL95-2. We obtained the mean counts per million (CPM) value for each miRNA in JAr EVs and the mean log2FC of downregulated putative target genes for each miRNA. We then performed a weighted Pearson’s correlation using the R package ‘weights’ (https://cran.r-project.org/web/packages/weights/index.html),whereby each miRNA was weighted according to the number of downregulated targets (Fig. 1).

### Experimental design

#### Investigating the RNA cargo of extracellular vesicles

JAr and HEK293 cells were cultured and spheroids were formed according to the methods and conditions described above. Approximately 1 × 10^5^ spheroids were prepared from each cell type. Once the spheroids were fully formed, they were transferred into 60 mm dishes containing 5 ml EV depleted culture media (5,000 spheroids per dish). Spheroids were incubated in a slow rotating gyratory shaker for 24 h to stop the spheroids from losing the structural cohesion. After incubation, conditioned media (approximately 100 ml) were collected and EVs were isolated. After removing the EVs used for supplementation, remaining EVs (approximately 1 × 10^12^ EVs per each sample) were subjected to RNA extraction and mRNA sequencing was performed. Samples were prepared in three different days for EV supplementation and mRNAseq. Samples used for miRNA sequencing were prepared separately and a minimum of 1 × 10^7^EVs were used for miRNA sequencing for each sample in three biological replicates for each group.

### Determining the effects of JAr and HEK293 cell derived EVs on RL95-2 cellular transcriptome

Endometrial analogue (RL95-2) cells were cultured in 12 well plates until 80% confluency using the culture methods and conditions described above. At the desired confluency, growth media was removedand 1 × 10^8^ EVs derived from trophoblast analogue (JAr) and non-reproductive cellular spheroid (HEK293) cells, were added to the RL95-2 cell monolayer separately in an EV-depleted supplementation media. Controls were prepared using untreated RL95-2 cells cultured in EV-depleted media. Cells were incubated for 24 h. After incubation, the media was removed and cellular RNA was collected for sequencing. The experiment was performed on three different days to prepare the three biological replicates.

## Results

### JAr cell derived EVs induced significantly differentiated gene expression in RL95-2 cells while HEK293 cell derived EVs failed to induce a similar effect

**Fig. 2:**
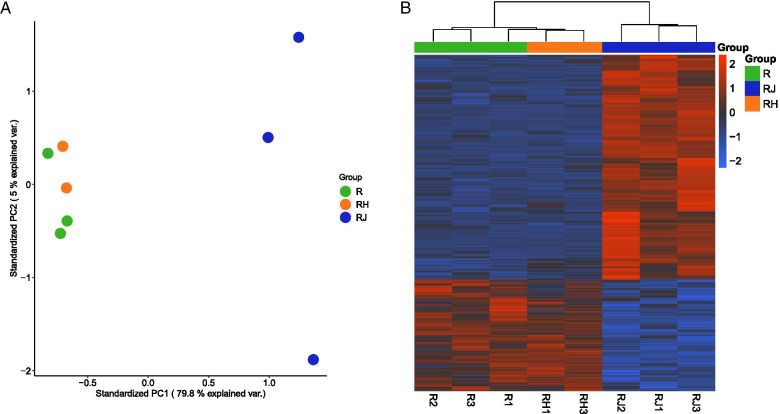
Gene expression profile of RL95-2 cells supplemented with JAr EVs and HEK293 EVs. (**A**) Principal component analysis (PCA) of all genes considered expressed in either of the three groups: RL95-2 cells supplemented with JAr EVs (RJ), RL95-2 cells supplemented with HEK293 EVs (RH), and un-supplemented control RL95-2 cells (R). The first two principal components (PC) are presented. (**B**) Heatmap and unsupervised hierarchical clustering (Euclidean distance) of the 1,787 differentially expressed genes. (DEGs) in the RJ vs R comparison

JAr cell spheroid-derived EVs and HEK293 cell spheroid-derived EVs were supplemented to RL95-2 cell monolayers separately and incubated for 24 h. Control samples were prepared using untreated RL95-2 cells. After incubation, the cellular RNA was extracted and sequenced for mRNA expression. Differential expression was calculated with reference to untreated control (R). The expression profile of RL95-2 cells treated with JAr spheroid derived EVs (RJ) was clearly different from the untreated control (R) and the RL95-2 cells treated with HEK293 spheroid-derived EVs (RH) (Fig. 2A). One of the RH samples was excluded from this analysis as an outlier (supplementary Figure 1). Nevertheless, it is apparent that the untreated RL95-2 cells and RL95-2 cells treated with HEK293 EVs are clustered relatively closely together, indicating that there was little or no effect on RL95-2 cells from HEK293 derived EVs. Differential expression (DE) analysis of the RJ and R group yielded 1166 upregulated and 588 downregulated genes in RJ group compared to the untreated RL95-2 cells.Similar analysis comparing R and RH group did not yield any significant differential expression. The similarity between the R and RH groups is abundantly clear based on the expression levels of aforementioned DEGs (Fig. 2B). The results of DE analysis suggest that JAr spheroid derived EVs can induce significant changes in RL95-2 transcriptome while the HEK293 derived EVs lack that capability.

### Gene set enrichment analysis of JAr EV-targeted genes in RL95-2

The most significantly enriched pathways in RL95-2 cells supplemented with JAr EVs are listed in the Table 1.

**Table 1:** Results of GSEA based on the differential expression analysis of RL95-2 cells treated with JAr EVs

Reactome ID	Description	NES	FDR
R-HSA-372790	Signalling by G-protein coupled receptor (GPCR)	1.267	0.010
R-HSA-388396	GPCR downstream signalling	1.289	0.010
R-HSA-1474228	Degradation of the extracellular matrix (ECM)	1.479	0.010
R-HSA-1474244	Extracellular matrix organization	1.448	0.010
R-HSA-1474290	Collagen formation	1.490	0.010
R-HSA-216083	Integrin cell surface interactions	1.549	0.010
R-HSA-3000171	Non-integrin membrane-ECM interactions	1.456	0.011
R-HSA-3000157	Laminin interactions	1.568	0.019
R-HSA-3000178	ECM proteoglycans	1.441	0.051

Results of GSEA based on the differential expression analysis of RL95-2 cells supplemented with EVs compared to non-supplemented control group RL95-2 cells. Normalized Enrichment Score (NES) and False Discovery Rate (FDR) are presented

Two major pathways, ECM organization (R-HSA-1474244) and signalling by GPCR (R-HSA-372790) were significantly affected by JAr EVs effects on RL95-2 cells. Other significantly affected pathways are events of the two major pathways, for instance, the pathway GPCR downstream signalling (R-HSA-388396) is one of the two first level events of the signalling by GPCR pathway. Other significantly enriched pathways indicated in the table are events of ECM organization (R-HSA-1474244) pathway, such as collagen formation (R-HSA-1474290). The net positive Normalized Enrichment Score (NES) values of these pathways indicate that the genes involved in them were mostly upregulated in RL95-2 cells supplemented with JAr EVs, suggesting that the pathways themselves could also have been induced as the result of EV supplementation.

### JAr spheroid derived EVs carry distinct mRNA cargo compared to HEK293 cell derived EVs

**Fig. 3:**
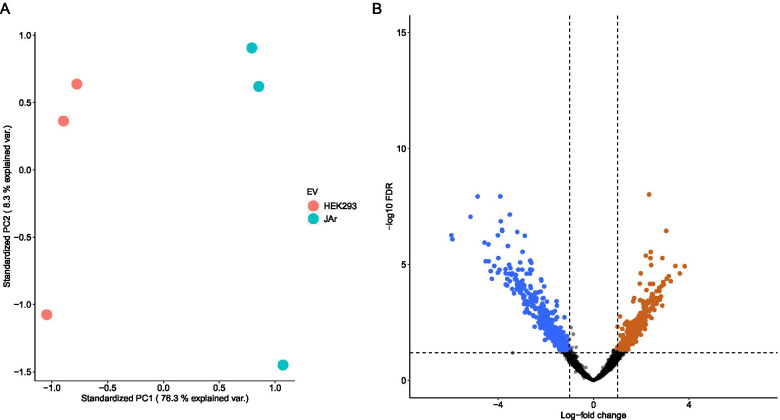
The contrast between RNA cargo of JAr EVs and HEK293 EVs. (**A**) Principal component analysis (PCA) of all genes for which RNA fragments were detected in the total RNA of either the JAr EVs (JAr) or HEK293 EVs (HEK293). The first two principal components (PC) are presented. Clear separation of HEK293 samples from the JAr samples can be seen along the PC1 axis. (**B**) Differential enrichment of RNA fragments aligning to known gene loci in the JAr EVs and HEK293 EVs. Genes for which the RNA fragments in JAr EVs were enriched compared to HEK293 EVs (FDR ≤ 0.05, log2FC > 1) are coloured orange. Genes for which the RNA fragments in JAr EVs were depleted compared to HEK293 EVs (FDR ≤ 0.05, log2FC < -1) are coloured blue

EVs were isolated from JAr and HEK293 spheroid conditioned media using size exclusion chromatography. EV RNA was extracted and the mRNA cargo of the EVs were sequenced. Enrichment of mRNA was calculated by contrasting the abundance of JAr EV mRNA to the abundance of HEK293 EV mRNA using edgeR as previously described. The population of RNA fragments aligning to known genes was substantially different between the JAr EV RNA and HEK293 EV RNA (Fig. 3A). 400 genes were found to be significantly enriched among the EV RNA fragments at logFC > 1and FDR ≤ 0.05 in JAr EV while 501 mRNA were significantly depleted at logFC < 1 and FDR ≤ 0.05 compared to HEK293 EV (Fig. 3B). Based on these data, the mRNA cargo appears to be significantly dependent on the type of cells producing the EVs.

### JAr spheroid derived EVs carry distinct miRNA cargo compared to HEK293 cell derived EVs

**Fig. 4:**
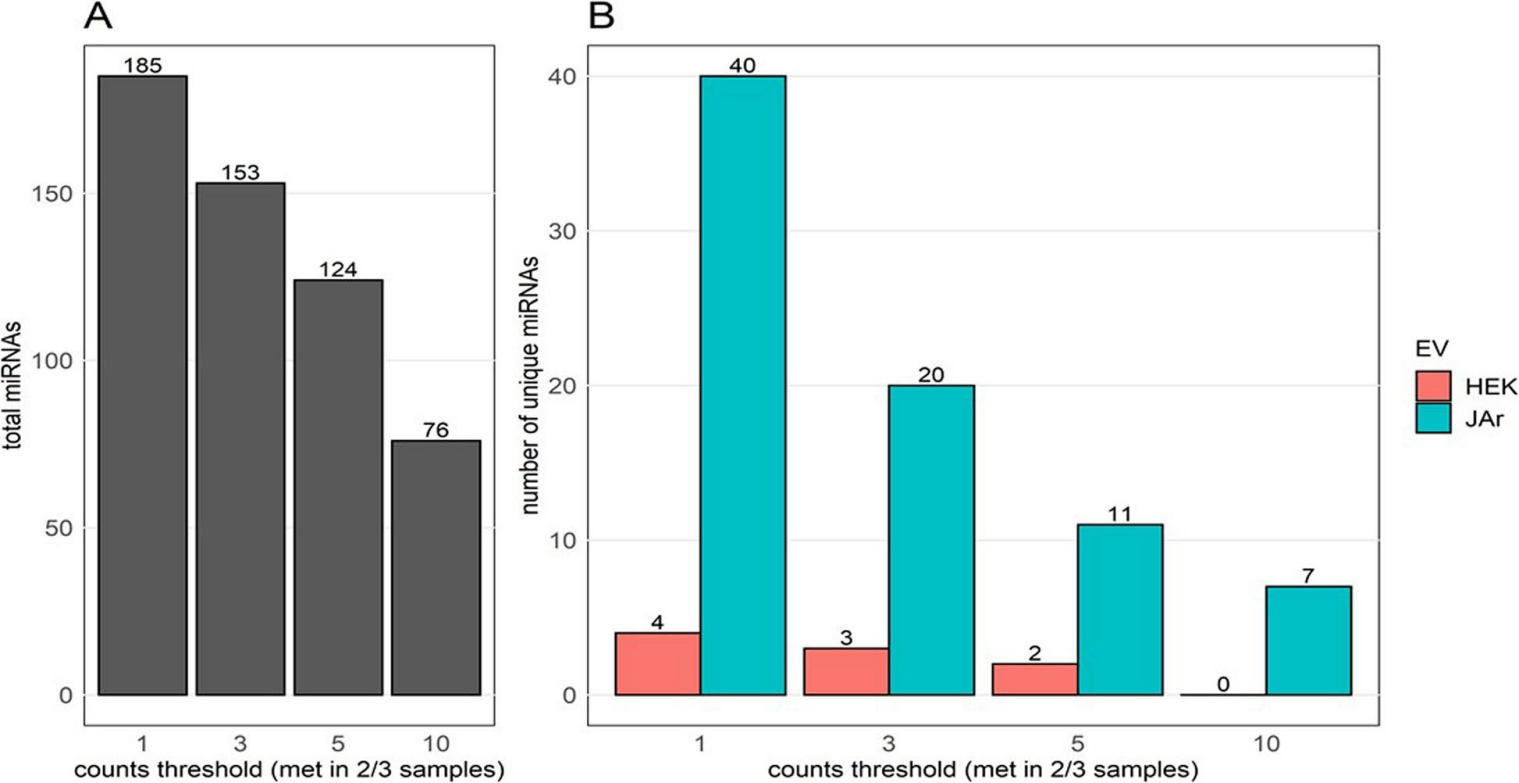
miRNA content of JAr and HEK293 EVs. Number of miRNAs detected in at least 2/3 libraries of one of the two EV types at four raw counts thresholds, i.e., at count’s threshold of 10 each miRNA needs to be counted at least 10 times in 2/3 libraries of either HEK293 or JAr EVs(A). Numbers of miRNAs considered to be unique to HEK293 or JAr EVs after passing four raw counts thresholds in at least 2/3 libraries of one of the two EV types(B). miRNAs were considered unique if they passed the required counts criteria for one EV type but were not detected at all in any of the libraries of the other EV type

JAr EVs were also distinguishable from HEK293EVs according to their miRNA content. The miRNA filtering criteria used for the analysis influenced both the total number of miRNAs detected in either of the two EV types examined (Fig. 4A) and the number of miRNAs which were unique to either JAr or HEK293 EVs (Fig. 4B). When considering a read count threshold of five which had to be met in 2/3 libraries within a given group (JAr or HEK293), 11 microRNAs were detected only in JAr EVs while only two were detected in HEK293 EVs. These 11 microRNAs were subsequently retained for further analysis of their target genes. miRNA abundance in JAr EVs correlates with fold change of downregulated target genes in RL95 cells.

**Fig. 5:**
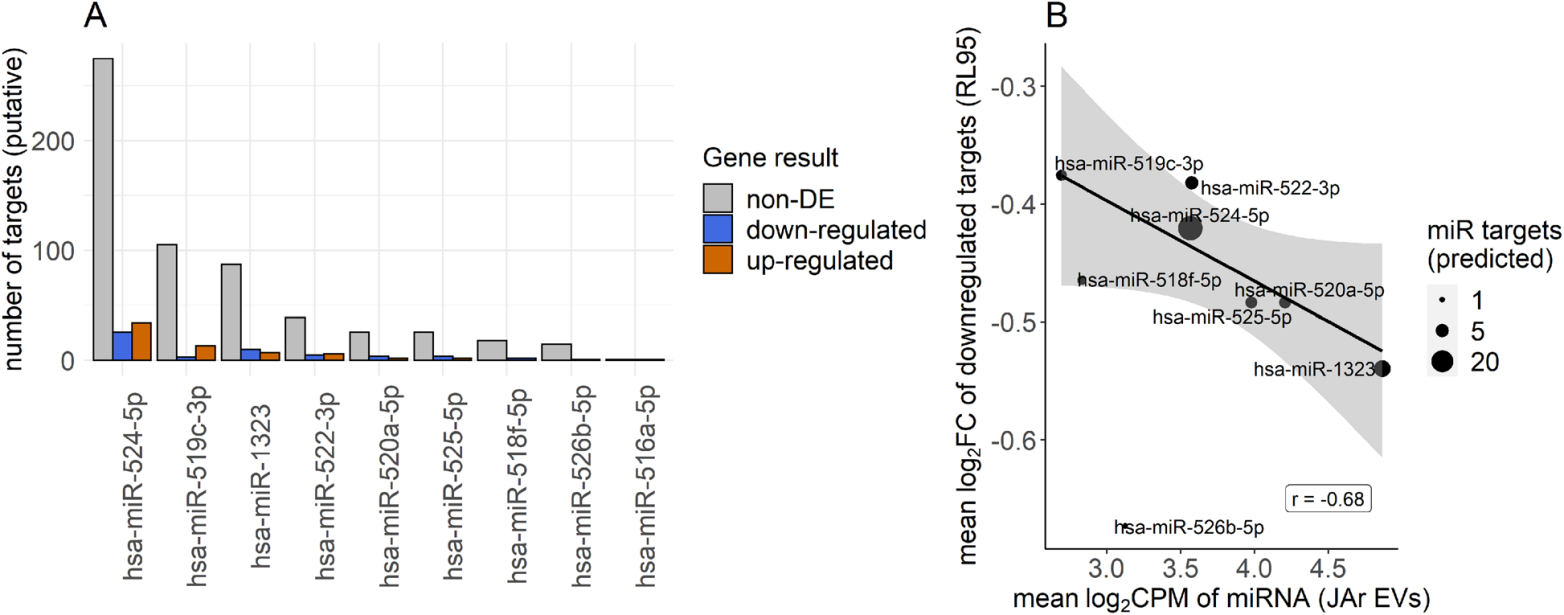
(**A**) Eleven miRNAs identified as specific to JAr EVs and their corresponding numbers of putative high-confidence (miRDB target score ≥ 90) gene targets present in the RL95 gene expression dataset. Numbers of non-differentially expressed (grey bars), downregulated (blue) and upregulated target genes (orange) are shown. (**B**) Relationship between abundance of JAr-specific miRNA in JAr EVs (expressed as mean log2cpm, derived from three libraries) and the mean log2FC of downregulated (FDR ≤ 0.05) putative high-confidence targets (target score ≥ 90) in RL95 cells. The number of downregulated putative targets for each miRNA is represented by the point size. Weighted regression line (weighted by number of downregulated targets) with 95% confidence intervals of the mean are shown

For the 11 JAr-specific miRNAs, a total of 1,188 high-confidence putative gene targets were identified from miRDB database and applying a target score cut-off of 90. Of these, 744 were present within the RL95 gene expression dataset. Only a small proportion of these genes were differentially expressed, with 53 of them downregulated and 68 of them upregulated.Although more putative targets were upregulatedthan downregulated,putative miRNA targets constituted a higher percentage of total downregulated genes (9%) compared to both total upregulated(5.8%) and total non-differentially expressed genes (6.4%). Furthermore, six out of the eleven miRNAs had a greater number of targets which were downregulated than upregulated, while only four had a greater number of upregulated than downregulated targets (Fig. 5A). hsa-miR-524-5p had the largest number of putative targets represented in the expression dataset, the 26 downregulated targets of which constituted 4.4% of the total downregulated genes.

Although the number of miRNAs examined was low, the mean log2FC of downregulated target genes displayed a moderate negative correlation with the abundance of a given miRNA in JAr EVs (weighted Pearson’s correlation, r = -0.65, *p* = 0.041; Fig. 5B). The most abundant JAr-specific miRNA was has-miR-1323, the downregulated targets of which had the lowest log2FC of all miRNAs except for hsa-miR-526b-5p, which had only one downregulated target.The negative correlation was found to be influenced both by the target score cut-off and FDR cut-off used to detect downregulated genes, with no such correlations evident at more relaxed criteria (supplementary Figure 2).

We also examined whether any downregulated genes constituted high-confidence predicted targets of multiple miRNAs. In this regard, we found only twodownregulated genes that were the putative targets of at least three JAr-specific miRNAs: *ATF2* (predicted target of hsa-miR-524-5p, hsa-miR-520a-5p, and hsa-miR-525-5p) and *SPTSSA* (predicted target of hsa-miR-524-5p, hsa-miR-526b-5p, and hsa-miR-1323), respectively.

Target prediction analysis was not carried out for the transcriptome of RL95-2 cells treated with HEK293 derived EVs because there was no significant differential expression observed.

## Discussion

EVs are known to transport various molecules between cells and alter the biochemical and physiological state of the target cells [44–46]. Despite the increasing popularity and the ever increasing knowledge of EV-mediated intercellular communication [47], the specificity of the effect induced by EVs on target cells and the mechanism of EV-mediated communication is not yet well understood. In the current study we endeavoured to study the effect of JAr spheroid derived EVs (an analogue for pre-implantation embryo) and HEK293 spheroid derived EVs (a cell line of non-reproductive origin) on RL95-2 cells, analogue of WOI-status endometrium.

JAr EVs induced substantial alterations to the RL95-2 cells’ transcriptome. Interestingly, EVs derived from a non-reproductive cell line, HEK293, failed to induce a similar profile of differential expression in the RL95-2 cells (Fig. 2), thus demonstrating that trophoblast-like-cell derived EVs are uniquely capable of altering the transcriptome of endometrial cells.The specificity of EVs is a topic of intense scientific discourse [48]. Some reports [49, 50] posit that EVs are highly targeted and will only act on a specific type of cell or a tissue. However, other reports [51–53] suggest that EVs are indiscriminately uptaken by any type of the cells. In the current study we have demonstrated that trophoblast-like cell derived EVs exhibit functional specificity. Studies on functional specificity of EVs are conducted, to a large degree, in the field of cancer metastasis where EVs derived from cancerous cells are reported to regulate the reaction of target cells in metastasis [54, 55]. To the best of our knowledge, this is the first report of functional specificity of EVs in the context of embryo-maternal communication. This compelling effect could be attributed to either the selective uptake of JAr-EVs, when compared to HEK293 derived EVs by RL 95–2 cells or to the differences between the EV cargo of JAr and HEK293 EVs. Since in this study the JAr/RL95-2 model was used to mimic the pre-implantation uterine microenvironment, we could deduce that the transcriptomic changes induced by EVs are not random, but specific to embryo derived EVs and have functional significance for embryo-maternal communication.

The probable functional importance of the JAr EVs’ effect on RL95-2 cells is more apparent when considering the pathways affected by the DEGs (Table 1). For example, the majority of the events of the extracellular matrix (ECM) organization pathway (R-HSA-1474244) were tagged by the genes upregulated in the RL95-2 cells treated with JAr EVs. ECM remodelling is a critical morphological and biochemical alteration the endometrium undergoes in preparation for implantation. It promotes and stabilizes the embryo adhesion while protects the underlying stromal cell layer from over invasion by the extravillous trophoblasts [56–58]. Major components of the pathways, such as laminin interactions (R-HSA-3000157) [59, 60], integrin cell surface interactions (R-HSA-216083) [61–66], and non-integrin membrane-ECM interactions (R-HSA-3000171) [67] are all known to be implicated in endometrial modifications in the WOI.

The pathway of signalling by GPCRs (R-HSA-372790) was also found to be significantly enriched by DEGs. GPCRs are the largest family of transmembrane receptors accounting for 4% of the coding regions of the human genome [68]. They are known to bind a highly diverse set of ligands that perform biological functions ranging from sight and olfactory senses to immune regulation [69, 70]. They also act as receptors for a number of ligands known to alter the endometrial microenvironment during the WOI [71], such as hCG [72], prostaglandin E2 [73, 74], cytokines [75–77] and progesterone via its membrane bound receptor [78–80]. Downstream signalling of GPCR pathway (R-HSA-388396) is also significantly enriched by the DEGs. These downstream pathways are secondary messengers that modify the endometrial morphology to facilitate implantation. For example, phosphatidylinositol 3-kinase/protein kinase B (PI3K/Akt) which regulates cell growth and survival, is reported to be involved in endometrial cellular migration, which is crucial in embryo attachment and invasion [81–84]. Evidence from the top pathways resulting from GSEA leads to the notion that the transcriptomic changes induced by JAr EVs on RL95-2 cells are not only directly modifying the endometrium for imminent implantation, but also modifying and priming the endometrial membrane receptors for further reception of embryonic signals, such as hormones and cytokines.

We investigated the RNA contents of the EV populations derived from JAr and HEK293 cells to collect data about the mechanism of EV induced transcriptomic changes. Identification of the RNA cargo of the two types of EVs by sequencing mRNA and miRNA was the first step of the investigation. There were significant (FDR ≤ 0.05) differences between mRNA found in JAr and HEK293 EVs indicating that the two EV populations are distinct from each other (Fig. 3). However, we were not able to confirm any connection between the observed transcriptomic alterations and the mRNA cargo of EVs.

In addition to mRNA, JAr EVs differed from HEK293 EVs in their microRNA composition. miRNAs are regulators of gene expression which uses multiple mechanisms to inhibit, destabilize and cleave transcripts [85–87]. There are about 1115 miRNAs identified and characterized which target about 60% of the human genes [88–90]. It is well-documented that miRNAs play important roles in cell-to-cell communication [91–93] and numerous studies have further demonstrated the role of EVs in transporting miRNAs to facilitate intercellular signalling. When applying a reasonable counts threshold (5 counts in 2/3 libraries of either JAr or HEK293), we identified eleven microRNAs in JAr EVs which were not detected in HEK293 EVs. Interestingly, while substantially more JAr-specific miRNAs could be detected while applying lower counts thresholds, relaxing the counts threshold did not substantially influence the number of HEK293-specific miRNAs in EVs, suggesting that the majority of miRNAs present in HEK293 EVs are indeed also present in JAr EVs. We reasoned that the 11 microRNAs could be considered to be JAr-specific and were retained for downstream target analysis. Although the ability to detect differences between the miRNA profiles of different EV types is highly influenced by technical factors such as read depth and filtering criteria [94–98], multiple reports nevertheless corroborate the presence of contrasting miRNA profiles in populations of EVs isolated from different cell types and biological fluids [23, 99, 100]. These differences, if present, could aid in interpreting the observed EV induced effects on RL95-2 cellular transcriptome.

Although we hypothesized that some gene expression changes may be putatively linked with miRNAs present in JAr EVs, the finding that the majority of gene expression changes constituted upregulation suggests that canonical miRNA-induced silencing is not the primary mode of action by which JAr EVs modulate gene expression changes in target cells. Indeed, we believe that the 11 microRNAs identified as JAr-specific had high-confidence putative targets among both downregulated and upregulated genes in RL95-2 cells. Moreover, in the absence of any miRNA-induced silencing, we would expect the relative proportions of downregulated and upregulated putative targets to reflect the relative proportions of overall up- and down-regulation of genes, which was not the case in our results. Indeed, most JAr-specific miRNAs had more high-confidence predicted targets that were downregulated than upregulated, and miRNA targets constituted a higher proportion of downregulated genes compared to either upregulated or non-DE genes. Furthermore, the log2FC of downregulated putative targets negatively correlated with the abundance of 11 miRNAs detected in JAr EVs, with the putative targets of the most abundant JAr-specific miRNA (hsa-miR-1323) showing the strongest decrease in mean log2FC. Collectively, these observations suggest that at least some of the downregulation may have been a direct result of canonical miRNA silencing. We also note that, as we co-incubated EVs with RL95-2 cells for 24 h, we cannot exclude the possibility that at least for some of the increased expression levels were caused by the secondary effects of EV-derived miRNAs.

The majority of the observed DEG’s cannot be explained as a direct effect resulting from either mRNA or miRNA transported to the recipient cells via EVs. Considering the relatively small copy number of RNAs transported in EVs, attributing the substantial degree of DE to direct effects of EV RNA on target cells would be illogical [101, 102]. However, based on the results of the current study, the role played by EVs, and specifically JAr-derived EVs as the mediator of the effect is clear, even though the molecular mechanisms underlying the majority of the transcriptomic changes remain elusive. It can be speculated that other types of regulatory mechanisms such as biomolecules bound to the EV surface, other non-coding RNAs, transcription regulation enzymes or cytokines carried in the EVs could play a role in the observed effects on endometrial transcriptome.

The phenomenon of functional specificity of EVs was clearly demonstrated in our experiments. However, it remains unclear to what extent the observed differences in RNA cargo between the two EV types (JAr and HEK293) can account for the transcriptomic changes observed in the target cells. It might be possible to reveal a clearer picture of this form of intercellular communication if this effect would be measured in detailed series of time points.Also, future studies would be required to explore the issue of EVs’ functional specificity via other avenues of investigations, such as the degree of uptake specificity exhibited by the RL95-2 cells towards trophoblast-like-cell and non-trophoblast spheroid derived EVs,the surface proteome differences between the two types of EVs which could play a role in membrane-based intercellular signalling, differences in EV cargo other than regulatory RNA, the mechanisms employed by the target cells to interpret the signals delivered by EVs and the target specificity of trophoblast spheroid derived EVs in terms of target cells of reproductive and non-reproductive origin.

## Conclusions

We have shown that trophoblast-like-cell derived EVs were capable of inducing unique transcriptomic alterations in target endometrial cells. These changes were only induced by trophoblast-like-cell derived EVs, implying functional specificity of these EVs.Some of the observed changes, such as those associated with extracellular matrix remodelling and GPCR mediated signalling, may be indicative of functional components of embryo-maternal communication in implantation.This effect was unique to the trophoblast-like-cell derived EVs compared to EVs from a non-reproductive source, which may be at least partly due to the distinct RNA cargo of the EVs from the two sources. However, as the RNA composition of JAr EVs could be putatively linked with only a small proportion of gene expression changes, future studies should aim to explore the role of different molecular pathwaysunderlying EV-mediated transcriptomic changes, as well as further elucidate their functional role in embryo-maternal communication.

## Supplementary Information

**Additional file 1: **Supplementary** Figure S1:  **Data pertaining to the outliers observed in the RNAseq analysis.

**Additional file 2:** Supplementary **Figure S2**: Data pertaining to the variation of the correlation between mean log2FC of downregulated target genes with the abundance of a given miRNA in JAr EVs in different levels of target prediction criteria.

## Data Availability

The sequencing data has been uploaded to the NCBI SRA repository (www.ncbi.nlm.nih.gov/sra) under the accession number PRJNA692831.

## Abbreviations

EV: Extracellular Vesicle
FBS: Foetal Bovine Serum
RNAseq: RNA Sequencing
DE: Differentially Expressed
DEG: Differentially Expressed Genes
FDR: False Discovery Rate
GSEA: Gene Set Enrichment Assay
PCA: Principal Component Analysis
GPCR: G Protein-Coupled Receptors
ECM: Extracellular Matrix
PC: Principal Component
