# Supplementary figures and images for: Trophoblast derived extracellular vesicles specifically alter the transcriptome of endometrial cells and may constitute a critical component of embryo-maternal communication
Source: Reprod Biol Endocrinol. 2021 Jul 21;19:115. doi: 10.1186/s12958-021-00801-5 (PMC8293585; doi:10.1186/s12958-021-00801-5)

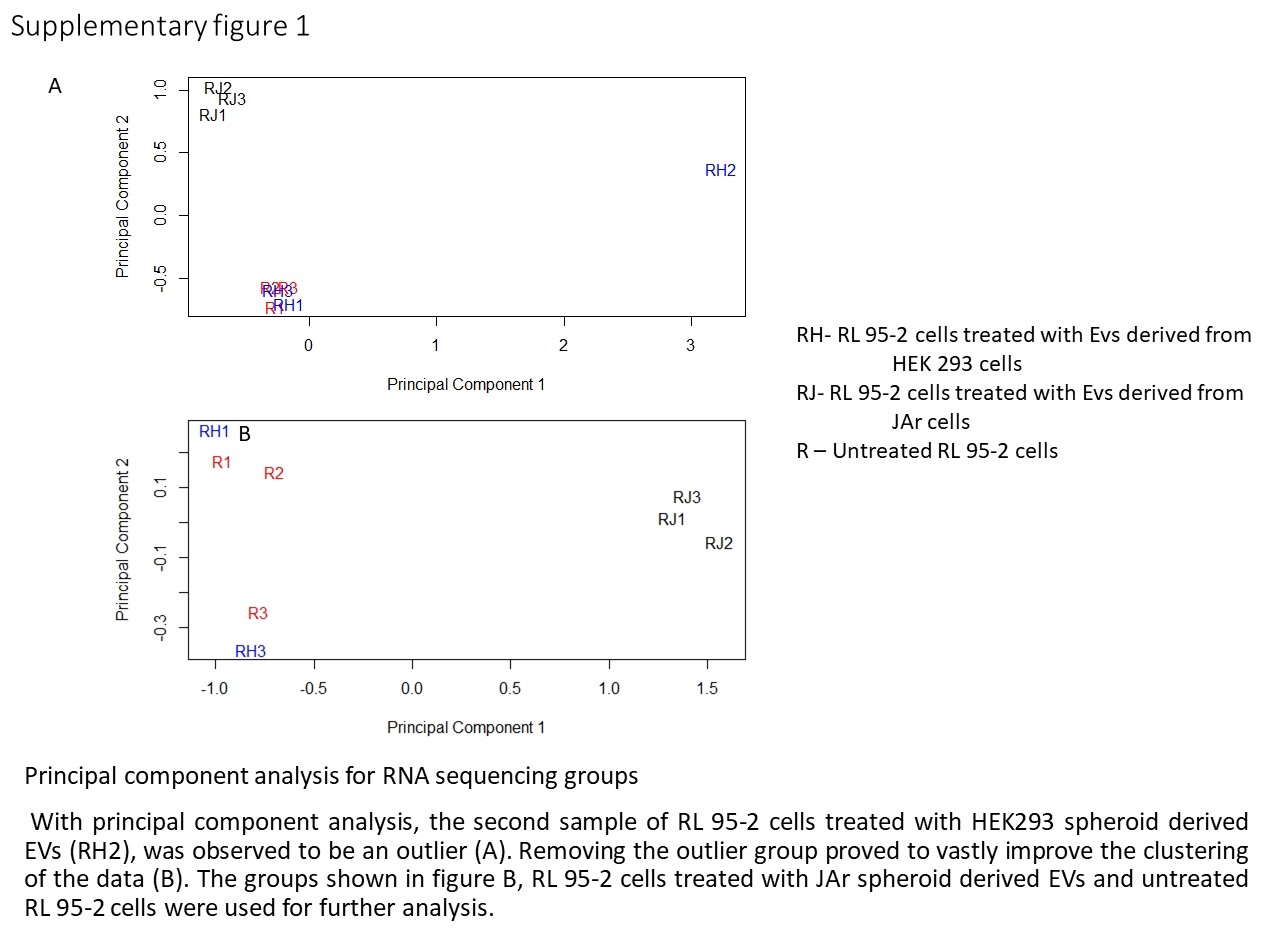

Supplement: Supplementary file 1 — Additional file 1: Supplementary Figure S1: Data pertaining to the outliers observed in the RNAseq analysis. [file 12958_2021_801_MOESM1_ESM.jpg]

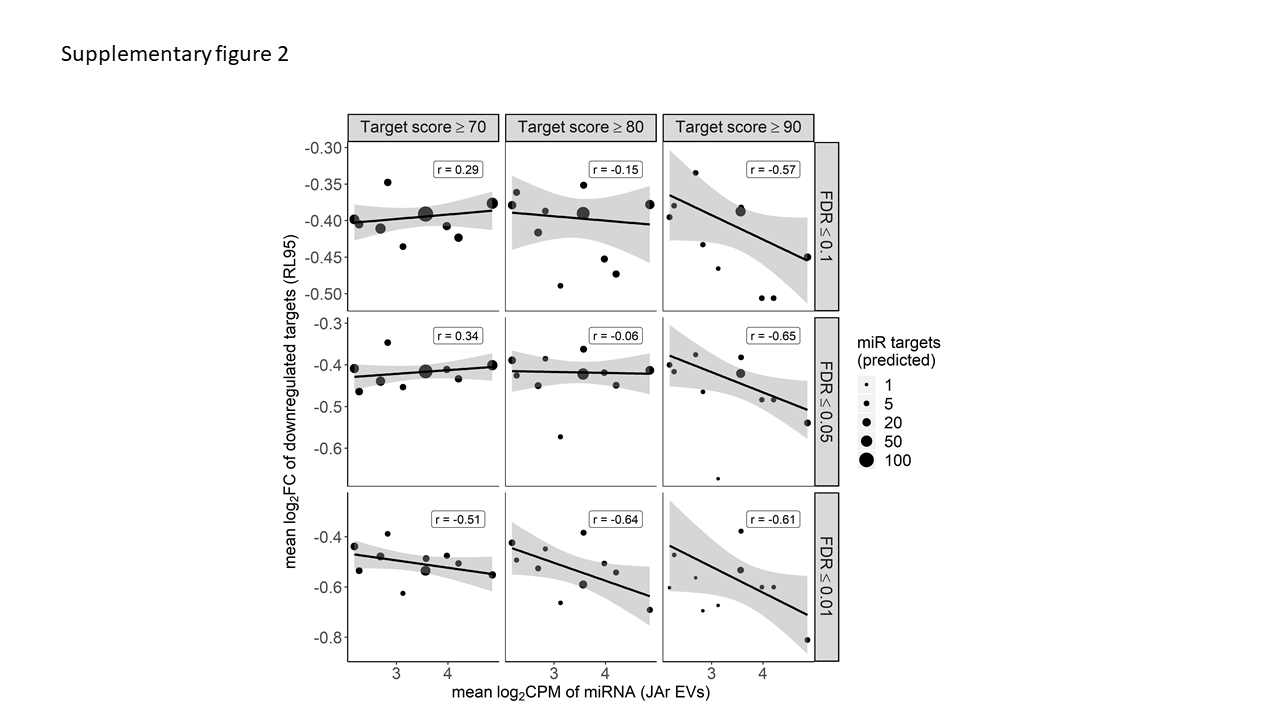

Supplement: Supplementary file 2 — Additional file 2: Supplementary Figure S2: Data pertaining to the variation of the correlation between mean log2FC of downregulated target genes with the abundance of a given miRNA in JAr EVs in different levels of target prediction criteria. [file 12958_2021_801_MOESM2_ESM.png]
